# Establishing paediatric reference intervals for thyroid function tests in Croatian population on the Abbott Architect i2000

**DOI:** 10.11613/BM.2021.030702

**Published:** 2021-08-05

**Authors:** Adriana Bokulić, Ivana Zec, Domagoj Marijančević, Sanja Goreta, Marija Požgaj Šepec, Lavinia La Grasta Sabolić

**Affiliations:** 1 Laboratory of Endocrinology, Department of Oncology and Nuclear Medicine, Sestre Milosrdnice University Hospital Center, Zagreb, Croatia; 2 Department of Paediatrics, Sestre Milosrdnice University Hospital Center, Zagreb, Croatia

**Keywords:** paediatric, reference values, thyroid function tests, immunoassay, standard deviation ratio

## Abstract

**Introduction:**

Evaluation of thyroid function is often requested and therefore defining paediatric reference intervals (RIs) is of vital importance. Currently, there is a distinct lack of paediatric RIs for thyroid function tests in Croatia. Thus, we established RIs for thyroid stimulating hormone (TSH), total triiodothyronine (TT3), total thyroxine (TT4), free triiodothyronine (FT3) and free thyroxine (FT4) in the Croatian paediatric population.

**Materials and methods:**

Reference intervals were calculated from 397 apparently healthy children, aged from 2 days to < 19 years. Serum samples were analysed for thyroid function tests on the Abbott Architect i2000. Age- and sex-specific 95% RIs with 90% confidence intervals were established according to Clinical and Laboratory Standards Institute guidelines. To express the magnitude of sex and age variation, standard deviation ratio (SDR) was calculated using two-level nested ANOVA. The criterion for considering partitioning reference values was set to SDR > 0.3.

**Results:**

All thyroid function tests required age partitioning, confirmed by SDR above 0.3. There was no need for sex partitioning, confirmed by SDR below 0.3. Still, FT3 was partitioned due to visually noticeable sex related difference for the oldest group (12 years to < 19 years).

**Conclusion:**

This is the first study to establish RIs for thyroid function tests in the Croatian paediatric population. We propose RIs for widely used Abbott platform, thus giving laboratories method- and population-specific paediatric RIs for thyroid function tests that should improve clinical test interpretation.

## Introduction

Reference interval (RI) is commonly defined as range of values within two reference limits, including the limits themselves (*1*). It represents an important tool, which aids clinicians in diagnosing, making therapeutic decisions, monitoring, and generally in differentiation between healthy and non-healthy individuals. Establishing paediatric specific RIs, despite being a difficult and sensitive task, became an essential demand for appropriate patient management (*2*).

The assessment of thyroid function is the most frequently applied endocrine investigation in children and adolescents. An important aspect of thyroid function is the dynamic change that takes place during growth and development (*3*). Although interpretation of thyroid function tests is similar to that applied in adults, the RIs differ since they depend on the maturation of the hypothalamic-pituitary-thyroid axis. Adult RIs are often the only available ones when interpreting laboratory results for paediatric population. Relying solely on adult RIs could however lead to unnecessary diagnostic procedures and erroneous therapeutic decisions. Hence, it is essential to use appropriate age-related RIs and to interpret results based on a thorough knowledge of thyroid physiology from neonatal age to adulthood (*4*).

In the last decade, there were multiple published studies with RIs for thyroid hormones in children and adolescents (*5*). Differences in study design and method of measurement create difficulties when comparing these results. Furthermore, it is well known that the Abbott Architect method produces lower TSH results, which leads to lower TSH values in studies using this method (*6*-*9*). Even in studies using the same method, the differences are still present due to variability in subject recruitment (hospital patients *vs* healthy children), age- and sex- partitioning, ethnicity and iodine status of studied population (*10*-*14*).

Croatian laboratories are responsible for providing appropriate RIs for local population. Usually, they rely on literature data derived from other populations and verify them according to Clinical and Laboratory Standards Institute (CLSI) protocol before implementation (*1*, *15*). The concentration of thyroid hormones can be influenced by multiple factors other than age and sex, such as ethnicity, iodine status and body mass index (*16*-*18*). Currently, there is a distinct lack of paediatric RIs for thyroid function tests in Croatia.

We intended to establish RIs for thyroid function tests including thyroid stimulating hormone (TSH), total triiodothyronine (TT3), total thyroxine (TT4), free triiodothyronine (FT3) and free thyroxine (FT4) in the Croatian paediatric population. The method-specific RIs empirically determined in this study are expected to improve the quality of the immunoassay interpretation for paediatric care in many hospitals and clinical laboratories using the Abbott platform for their automated immunoassay testing.

## Materials and methods

### Subjects

The study population was prospectively recruited from October 2018 to July 2020 in the University Hospital Center Sestre milosrdnice, Zagreb, Croatia. The study was approved by hospital’s Ethics Review Board. In total, 444 apparently healthy children, 207 males and 237 females, younger than 19 years of age entered the study. Residual serum samples were collected from routinely ordered laboratory requests from paediatric outpatients and maternity ward. Subjects were recruited by two experienced paediatricians according to inclusion and exclusion criteria. Inclusion criteria were defined as: age < 19 years, personal medical history and clinical examination non-suggestive of thyroid disease. Subject were excluded if any of the following criteria have been met: premature birth (for neonates), established chronic disease, acute illness within 7 days of collection, intake of medications known to affect serum concentration of thyroid hormones, body mass index below 5 or above 95 percentiles for age and sex. Samples with thyroid peroxidase antibody (TPO-Ab) and thyroglobulin antibody (TG-Ab) results above manufacturer’s declared cut-off value of 5.6 kU/L and 4.1 kU/L, respectively, were excluded except for neonatal samples. Ideally, study should exclude samples with positive TPO-Ab and TG-Ab results, but this would significantly affect number of neonatal samples. Although diffusion of TPO-Ab through placental barrier is possible, the presence of TPO-Ab and TG-Ab in children does not affect estimation of RIs of thyroid function tests (*19*, *20*). All subjects fulfilling conditions of inclusion and exclusion criteria were defined as apparently healthy.

### Methods

Neonatal blood samples were collected regardless of the time in the day or fasting state by heel prick in micro tubes (0.5 mL MiniCollect, Greiner Bio-One GmbH, Kremsmünster, Austria). Other blood samples were collected in fasting state from 7.00 to 10.00 am by venipuncture in test tubes with clot activator (2 or 4 mL Vacuette, Greiner Bio-One GmbH, Kremsmünster, Austria). After clotting, blood samples were centrifuged at 2200 RCF for 10 minutes.

Fresh serum samples were firstly analysed for TPO-Ab and TG-Ab. If antibodies tested negative, samples were further analysed for TSH, TT3, TT4, FT3 and FT4. Neonatal samples were tested for all hormones irrespective of the antibodies test results. Due to limited volume, not all hormones were measured in each individual sample. All measurements were performed on the Abbott Architect i2000 chemiluminescent microparticle immunoassay (Abbott Diagnostics, Abbott Park, USA). Assay precision was evaluated according to CLSI EP15-A2 guidelines using two levels of quality controls measured in triplicate for five consecutive days (Table 1) (*21*). Prior to analysis, authorized personnel verified calibrations and quality controls.

**Table 1:** Assay precision results

		**Precision (CV%)** **Level 1**			**Precision (CV%)** **Level 2**	
**Analyte (unit)**	**Mean**	**Within run**	**Within laboratory**	**Mean**	**Within run**	**Within laboratory**
**TSH (mIU/L)**	0.017	3.9	4.5	3.48	1.4	1.8
**TT3 (nmol/L)**	1.1	2.7	3.0	2.6	3.5	4.0
**TT4 (nmol/L)**	68	2.5	2.9	138	3.1	3.0
**FT3 (pmol/L)**	2.7	4.5	5.2	8.5	2.4	3.5
**FT4 (pmol/L)**	10.9	2.3	2.4	28.7	2.8	2.5
TSH - thyroid stimulating hormone. TT3 - total triiodothyronine. TT4 - total thyroxine. FT3 - free triiodothyronine. FT4 - free thyroxine. CV - coefficient of variation.						

### Statistical analysis

Data collection and analysis were performed according to CLSI EP28-A3c guidelines (*1*). Hormone concentrations were plotted against age and sex. Plotted data were visually inspected for possible age and sex stratification and aberrant results. Shapiro-Wilk test was applied for testing normality. Outliers were tested using Tukey’s method and subsequently eliminated. Based on sample numbers (< 120 *vs* ≥ 120) and data distribution (normal *vs* non-Gaussian) in each subgroup, 95% RIs with 90% confidence intervals were calculated with appropriate statistical method as follows: (i) parametric - ≥ 120 samples with normal distribution; (ii) nonparametric percentile - ≥ 120 samples with non-Gaussian distribution; (iii) robust - < 120 samples with normal distribution or after Box-Cox transformation for non-Gaussian distribution. Method proposed by Ichihara and Boyd was used to evaluate the need for partitioning (*22*, *23*). The advantage of the nested ANOVA is to evaluate the magnitude of variation between three or more categories. Thus, we used the standard deviation ratio (SDR) for quantification of sex and age-related differences. In summary, two-level nested ANOVA was performed to calculate standard deviations (SD) of reference values for age (SD_Age_), sex (SD_Sex_) and SD attributable to net-between-individual variation (SD_BI_). Nested ANOVA computation was done after logarithmic transformation of reference values. To express the magnitude of each source of variation, SDR was calculated by dividing SD_Age_ and SD_Sex_ with SD_BI_. Criterion for considering partitioning reference values was set to SDR > 0.3. Results were provided through statistical software MedCalc version 19.2.1 (MedCalc Software Ltd, Ostend, Belgium) and Minitab version 19.2 (Minitab Statistical Software, AppOnFly Inc., San Francisco, USA).

## Results

**Figure 1:**
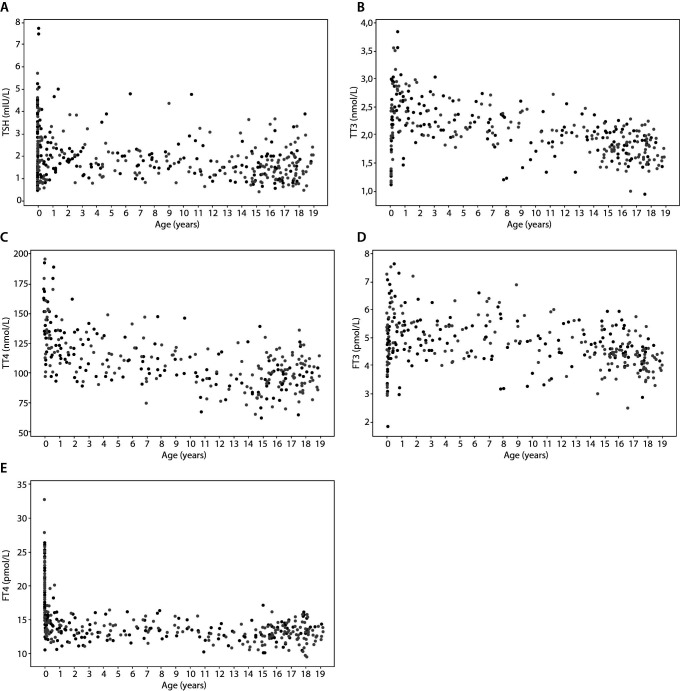
Age-specific scatter plots for thyroid function tests. Males are shown in black, females in grey. (A) TSH - thyroid stimulating hormone; (B) TT3 - total triiodothyronine; (C) TT4 - total thyroxine: (D) FT3 - free triiodothyronine; (E) FT4 - free thyroxine.

**Figure 2:**
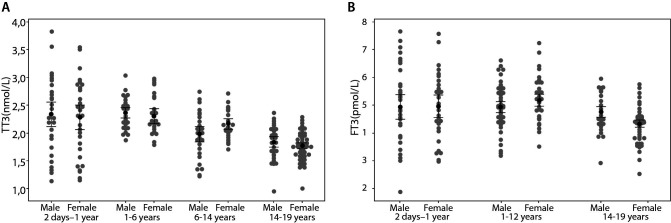
Interval plots by age groups. Values are presented as mean and 95% confidence intervals for (A) TT3 - total triiodothyronine and (B) FT3 - free triiodothyronine.

From a total of 444 subjects, 33 samples were excluded due to positive TPO-Ab and/or TG-Ab results. From 88 neonatal samples, 21 were TPO-Ab and/or TG-Ab positive, but were included in the study. After testing for outliers with Tukey method, 397 reference individuals, 187 boys and 210 girls, were selected for establishing RIs. Age- and sex-specific 95% RIs, 90% confidence intervals of the lower and upper reference limit and SDRs are presented in Table 2. Figure 1 represents scatter plots of thyroid function tests against age and sex. All RIs are defined beginning with 2-day-old new-borns, except TT4. The concentration of TT4 in the first days of life showed grossly scattered data that would affect upper reference limit and widen confidence interval. Since TT4 is not usually requested in new-borns, we decided to exclude results of new-borns younger than 7 days. Significant differences between age partitions were observed in all thyroid function tests with SDR_Age_ above 0.3 confirming partitioning data by age is appropriate and justifiable. Calculated SDR_Sex_ showed there is no need for sex stratification for any of the tested hormones. Figure 2 shows the interval plot for the only two analytes with measurable SDR_Sex_, TT3 and FT3. Even though SDR_Sex_ was less than 0.3, FT3 was partitioned due to noticeable sex related difference for the oldest group (12 years to < 19 years).

**Table 2:** Age- and sex-specific reference intervals with standard deviation ratios

					**Reference interval**				**Nested ANOVA**	
**Analyte (unit)**	**Age group**	**Sex**	**N**	**Median**	**Lower limit**	**Upper limit**	**Lower** **90% CI**	**Upper** **90% CI**	**SDR_Age_**	**SDR_Sex_**
**TSH (mIU/L)**	2 days to < 2 months*	M+F	102	2.28	0.50	6.54	0.41-0.64	5.71-7.35	0.34	0.00
	2 months to < 14 years^‡^	M+F	170	1.76	0.80	4.61	0.59-0.83	3.83-4.99		
	14 years to < 19 years^‡^	M+F	125	1.51	0.58	3.60	0.42-0.67	3.14 – 3.88		
**TT3 (nmol/L)**	2 days to < 1 year^†^	M+F	71	2.4	1.0	3.6	0.8-1.3	3.4-3.8	0.64	0.13
	1 year to < 6 years^†^	M+F	63	2.3	1.7	2.9	1.6-1.8	2.8-3.1		
	6 years to < 14 years^†^	M+F	66	2.1	1.4	2.8	1.3-1.5	2.7-2.9		
	14 years to < 19 years^†^	M+F	117	1.8	1.3	2.3	1.2-1.4	2.2-2.4		
**TT4 (nmol/L)**	7 days to < 1 year^†^	M+F	55	134	82	188	73-91	176-199	1.03	0.00
	1 year to < 9 years^†^	M+F	97	114	81	147	77-86	141-152		
	9 years to < 19 years^§^	M+F	148	97	68	126	64-71	123-130		
**FT3 (pmol/L)**	2 days to < 1 year^†^	M+F	74	4.8	2.4	7.4	2.0-2.8	7.0-7.8	0.80	0.21
	1 year to < 12 years^†^	M+F	113	5.0	3.5	6.6	3.3-3.7	6.3-6.8		
	12 years to < 19 years^†^	M	45	4.4	3.4	6.0	3.2-3.7	5.7-6.3		
	12 years to < 19 years^†^	F	85	4.4	3.2	5.5	2.9 - 3.3	5.3 - 5.7		
**FT4 (pmol/L)**	2 days to < 15 days^†^	M+F	68	19.8	11.8	28.0	10.3 - 13.1	26.4 - 29.6	1.64	0.00
	15 days to < 1 year*	M+F	54	14.2	11.3	18.9	10.8 - 11.8	17.7 - 20.1		
	1 year to < 19 years^§^	M+F	241	13.2	10.5	15.9	10.2 - 10.7	15.7 - 16.2		
TSH - thyroid stimulating hormone. TT3 - total triiodothyronine. TT4 - total thyroxine. FT3 - free triiodothyronine. FT4 - free thyroxine. M – male. F – female. *Robust method (Box-Cox transformation). ^†^Robust method. ^‡^Nonparametric percentile method. ^§^Parametric method. SDR_Age_ and SDR_Sex_ were computed by two-level nested ANOVA. SDR_Sex_ - standard deviation ratio for between-sex variations. SDR_Age_ - standard deviation ratio for between-age variations. CI – confidence interval.										

## Discussion

This study establishes age- and sex-specific RIs for thyroid function tests, derived from Croatian paediatric population using Abbott Architect method and confirms the necessity of establishing population specific RIs.

TSH is the first and the most important laboratory test used to evaluate thyroid function. As expected, surge in the concentration of TSH within first two months after birth and wider RIs are comparable to previous studies. With regard to method-matched group for the new-borns, only two studies reported RIs on the Abbott platform. The best agreement is noticed in the upper limit with Soldin *et al.*, which defined the same age group as our study (< 2 months) (*14*). Whereas the Canadian Laboratory Initiative on Pediatric Reference Intervals (CALIPER) study showed slightly lower upper reference limit, probably due to setting upper age limit to < 6 months (*12*). Most of the studies reveal significant reduction of TSH in adolescence in period between 11 and 15 years of age. The differences in the age groups could be from inconsistency in applied partitioning criteria (visual and statistical analysis *vs* Tanner stage *vs* arbitrarily divided age groups) (*10*-*14*, *24*, *25*). Visual inspection and statistical analysis of our data confirmed TSH partitioning at the age of 14. Regardless of different age groups, estimated RIs are similar with method-matched studies (*10*-*14*).

Our results showed TT4 and TT3 concentrations at the highest in the first year of life, then decreasing gradually towards adulthood. Interestingly, only Elmlinger *et al.* found moderate increase in TT4 around 7 years of age (*26*). Some authors reported significantly higher TT4 concentrations in females during adolescence (*12*, *20*). We did not find sex related differences, which is comparable only with Elmlinger *et al*. (*26*). Due to relatively small sample number in our study, the lack of gender effect on TT4 concentration should be explored on a larger sample size. As for TT3 in the eldest group, our data did not show sex related differences, which is consistent with two other studies (*25*, *26*). Even though the CALIPER study reported modest difference between sexes, our TT3 RIs are in best agreement with their results (*12*).

Throughout all age groups, our data showed slightly different FT3 concentration in lower reference limit, possibly due to population differences. The concentration of FT3 showed moderate changes after the first year until 11 to 14 years of age, where most other studies observed significantly higher concentrations in males (*10*, *12*, *27*). Sex differences in adolescents can be attributed to the effects of the oestrogens, growth hormone and insulin-like growth factor 1 on enhanced peripheral conversion (*28*, *29*). In contrast to other reports, our results showed that there were no sex related differences in adolescents with SDR_Sex_ < 0.3. However, visually plotted data showed shift to lower concentrations in adolescent females. This discrepancy could be the result of relatively small sample size, so we decided to separate adolescent FT3 RIs by sex. The FT3 concentration in adolescent are slightly lower both in lower and upper limit compared to some authors, but similar to the CALIPER eldest group (*10*, *12*, *13*, *27*, *30*).

FT4 concentration followed TSH surge in new-borns. Despite the fact that the same upper age limit at 15 days was used, our study showed significantly lower FT4 results than the CALIPER study (*12*). In contrast, Soldin *et al.* results were similar to ours, but with upper age limit set to 2 months (*14*). After the first year of life, FT4 remained stable until adulthood. Estimated RIs were similar to others within the Abbott group (*10*, *11*, *13*, *14*). Exception was the CALIPER study which used the same age group as ours (1 year to < 19 years) but found higher upper reference limit (*12*, *27*).

In general, previous studies reported significant changes in thyroid function tests after birth until adulthood. The current study results showed similar pattern with all thyroid function test concentrations being the highest after birth and gradually falling to adult concentrations. Regardless of differences in age partitioning between studies, our results are in close agreement with RIs for TSH in method-matched studies. In contrast, our RIs for other thyroid hormones showed significant shift in upper or lower limits. CALIPER Paediatric Reference Interval Database is one of most important databases for RIs in children and adolescents as it is based on a large sample of healthy population. This certainly eases the implementation of CALIPER based RIs in many laboratories. However, population differences do exist and require re-estimation of the same, as confirmed by this study results.

Reference intervals derived from this study are limited by the fact that samples were collected from hospital in- and out- paediatric patients rather than from healthy community children. In addition, samples were collected at only one hospital centre in the biggest central region of Croatia. Although Croatian population is homogenous, regional differences still may be present. Underlying diseases could influence the results despite our best efforts to exclude children with conditions potentially affecting thyroid hormones’ metabolism. Although the recommended number of 120 samples *per* group was not always achieved, appropriate statistical methods according to CLSI guidelines were used (*1*). We did not take into account body mass index and puberty stage which could influence RIs as suggested by some authors (*16*, *30*). Iodine status was not measured since Croatia is a country with sufficient iodine intake (*31*).

To our knowledge, this is the first study conducted on paediatric patients for thyroid function tests in Croatia. Partitioning RIs by age and sex was confirmed with calculated SDR from two-level nested ANOVA. We propose RIs for widely used Abbott platform, thus giving Croatian laboratories method- and population- specific paediatric RIs for thyroid function tests that should improve clinical test interpretation. Before implementing, laboratories still need to verify our proposed RIs based on CLSI protocol defined by C28-A3 guidelines (*1*).
